# Mendelian randomization provides causal association between COVID-19 and thyroid cancer: insights from a multi-cancer analysis

**DOI:** 10.3389/fonc.2024.1419020

**Published:** 2024-09-10

**Authors:** Shuhong Li, Zedong Du, Hui Ma, Liang Cai, Xiao Liu, Jie He

**Affiliations:** ^1^ Department of Oncology, Chengdu Second People’s Hospital, Chengdu, China; ^2^ Department of Gynecology and Obstetrics, West China Second University Hospital, Sichuan University, Chengdu, Sichuan, China

**Keywords:** Mendelian randomization, COVID-19, SARS-CoV-2, cancer risk, thyroid

## Abstract

Since the onset of the COVID-19 pandemic, the SARS-CoV-2 virus has caused over 600 million confirmed infections and more than 6.8 million deaths worldwide, with ongoing implications for human health. COVID-19 has been extensively documented to have extrapulmonary manifestations due to the widespread expression of necessary ACE2 receptors in the human body. Nevertheless, the association between COVID-19 and cancer risk remains inadequately explored. This study employs Mendelian randomization (MR) methods to examine the causal relationship between genetic variations associated with COVID-19 and the risk of developing cancer. The findings indicate that COVID-19 has negligible impact on most cancer risks. Interestingly, a higher COVID-19 impact is associated with a decreased risk of thyroid cancer. In summary, our findings demonstrate a genetic correlation between COVID-19 and thyroid cancer, contributing to our understanding of the interplay between COVID-19 and cancer risk.

## Introduction

Coronavirus Disease 2019 (COVID-19) is an infectious disease primarily characterized by respiratory symptoms caused by the SARS-CoV-2 virus. It rapidly spread worldwide after its initial reporting in 2019. As of now, there have been over 600 million confirmed cases and over 6.8 million reported deaths (1–3). Despite the highly contagious and harmful nature of the SARS-CoV-2 virus, not all individuals progress to severe illness. Studies indicate that approximately 81% of patients infected with the SARS-CoV-2 virus exhibit mild symptoms, 14% develop severe symptoms requiring hospitalization, and 5% progress to critical conditions requiring intensive care and respiratory support (4). Furthermore, the SARS-CoV-2 virus infects human cells through the angiotensin-converting enzyme 2 (ACE2) receptor, which is widely expressed in the human body (5). This infection leads to extensive extrapulmonary effects, including hematologic, cardiovascular, neurologic, and dermatologic systems, among others (6). Observations suggest that COVID-19 causes acute damage and functional abnormalities in these organs and tissue during the infection period, with long-term effects that require further observation (7).

Despite more than three years having passed since the beginning of the pandemic, research on the long-term effects of COVID-19 on humans is only just beginning. Follow-up studies on COVID-19 survivors have reported a series of long-term complications, including pulmonary abnormalities, endothelial damage, immune system dysregulation, and hypercoagulability, among others (7). However, the impact of cancer occurrence appears to have been overlooked in the assessment of numerous long-term effects, possibly due to the relatively short duration of the pandemic compared to the process of cancer development. The SARS-CoV-2 virus widely disseminates in the human body and persists for an extended period, which increases the risk of tumor occurrence in susceptible individuals (8–11). Limited studies have suggested that the SARS-CoV-2 virus might play a role similar to oncogenic viruses in the lungs and have emphasized the need for further research on the impact of COVID-19 on cancer (12, 13). In fact, virus-induced cancer occurrence is a common phenomenon in humans, including well-known viruses like the Epstein-Barr virus, hepatitis viruses, and human papillomavirus, which can cause carcinogenesis in multiple organs such as the blood, liver, reproductive system, skin, and more (14–16). Therefore, investigating the impact of COVID-19 on human cancer risk is a crucial research gap that needs to be addressed.

The development of cancer is a lengthy process, which poses challenges for existing observational data gathered from COVID-19 patients in generating robust conclusions regarding long-term cancer risk. Mendelian randomization (MR) can assist in overcoming this issue by examining genetic variation perspectives (17). MR uses randomly allocated genetic variants, known as Instrumental Variables (IV), to simulate the control of exposure factors in randomized controlled trials. Its goal is to obtain unconfounded estimates of the association between risk factors and outcomes, thereby avoiding potential residual confounding and reverse causation that observational studies might suffer from (18). Additionally, with the advancement of sequencing technologies, researchers have decoded extensive genomic sequences and mutations from various biological samples. Several large-scale Genome-Wide Association Study (GWAS) datasets have been shared, and analysis tools have been developed to facilitate the evaluation of the impact of SARS-CoV-2 virus on human cancer occurrence (18, 19). In our study, we employed MR analysis to assess the impact of three COVID-19 traits on the risk of 16 types of cancer and thyroid-related disorders in humans. We believe that this research enhances our understanding of COVID-19, particularly by providing new insights into the relationship between COVID-19 and human cancer risk.

## Methods

### Data source

This study utilized a total of 23 open-access Genome-Wide Association Study (GWAS) summary datasets, which included 3 datasets related to COVID-19, 16 datasets related to cancer. The summary data for all these datasets were obtained from the IEU OPEN GWAS PROJECT database (19). The selection criteria were based on incorporating the most recent, largest sample size, and openly accessible research data. The study numbers, along with detailed phenotype definitions, sample sizes, and other relevant information, have been provided in **Supplementary Table S1**.

### Filtration of IV

IV are tools used in mendelian randomization analysis to address endogeneity issues (20). They utilize genetic variations as instrumental variables to help infer causal relationships between observed variables (exposure factors) and outcomes. For each observed variable or exposure factor, genetic variations that are associated with the phenotype (p<5e-8) and are unrelated in terms of clustering and physical distance (r²<0.001, distance>10000kb) across the entire genome are selected (18). In the end, 17 Single Nucleotide Polymorphisms (SNPs) related to the three COVID-19 phenotypes were identified and used as IVs for further analysis in Mendelian randomization, as outlined in **Supplementary Table S2**.

### MR and sensitivity analysis

Two-sample MR analysis was employed to infer the causal associations between the exposure factors represented by IVs and various cancer outcomes. In this analysis, the three COVID-19 traits serve as exposure factors, and their associations with multiple cancer outcomes are the primary focus of the study design. The main evaluation method utilized in this study is the inverse-variance weighted (IVW) method, which is one of the most important methods in MR analysis (18). Finally, the risk contribution of the exposure factors to the outcomes is described using odds ratios (OR) and 95% confidence intervals (CI). Additionally, the effects of individual exposure SNPs on outcomes are evaluated using the Wald ratio method (18).

Due to the potential influence of pleiotropy in MR analysis, supplementary evidence is provided using the weighted median method (21) and MR-Egger regression (22). The MR-Egger method is employed to fit a linear regression to estimate intercepts and conduct statistical tests to assess the presence of pleiotropy. To evaluate the presence of heterogeneity in each design, Cochran’s Q test, funnel plots and MRPRESSO methods are utilized (23).

### Statistical analysis and visualization

The statistical analysis for this study was conducted using R software (Version 4.2.1) (24). The software package employed in the analysis process was “TwoSampleMR” (18). Forest plots were generated using the “forestploter” package (25). Visualizations such as scatter plots and funnel plots were created using the “TwoSampleMR” package. In this study, a significance level of 0.05 was utilized, meaning that we rejected the null hypothesis when the p-value was less than 0.05. To address non-independent multiple hypothesis testing, the False Discovery Rate (FDR) method was applied for multiple hypothesis correction, ensuring accurate control over the p-values.

## Results

### Identification of genetic IV

**Figure 1:**
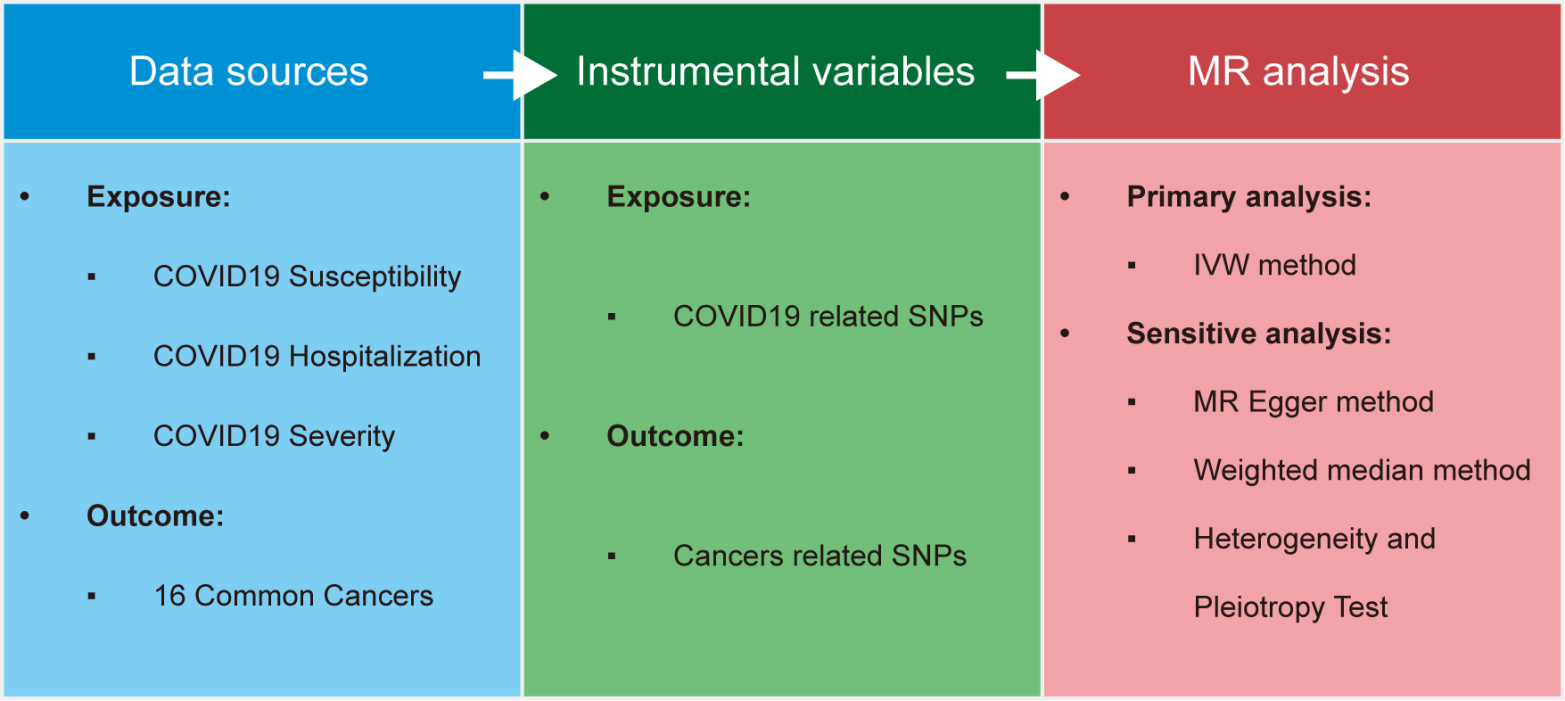
Workflow of this study.

Complete Analytical Approach of the Study as Illustrated in **Figure 1**. Firstly, we extracted a total of 17 SNP loci (**Supplementary Table S2**) closely associated with COVID-19 exposure from summary data of Genome-Wide Association Studies (GWAS) conducted in three distinct cohorts investigating COVID-19 patient susceptibility, hospitalization, and severity. Most of these loci were unique to each phenotype, with the exception of rs2109069 (**Supplementary Table S2**), which was common across all three phenotypes. Simultaneously, we collected summary data from GWAS studies conducted on 16 common human multi-site cancers. In these datasets, we identified 277 SNP loci most relevant to various types of cancer (**Supplementary Table S3**). The included study samples ranged from 182,625 to 1,887,658, with the number of cases ranging from 357 to 32,494. Detailed information about these cohorts is provided in the **Supplementary Materials** (**Supplementary Table S1**).

### Association between COVID-19 risk and cancer risk

**Figure 2:**
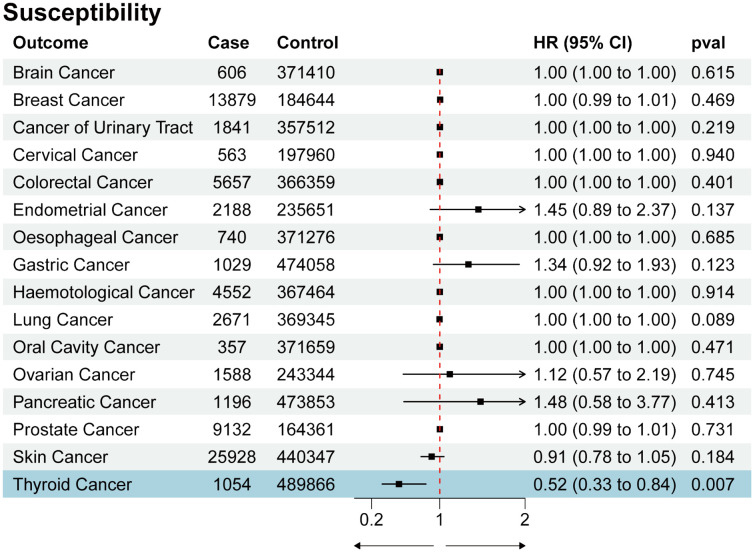
Forest plot of COVID-19 susceptibility effects on multi-cancer risk.

**Figure 3:**
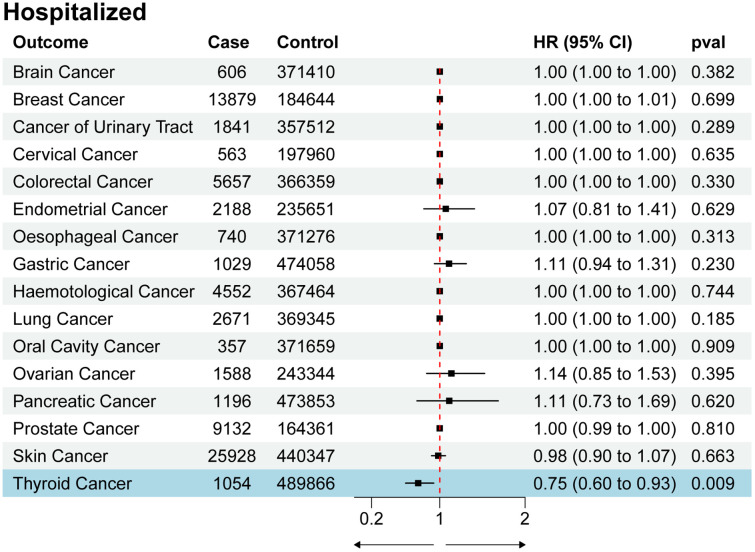
Forest plot of COVID-19 hospitalization effects on multi-cancer risk.

**Figure 4:**
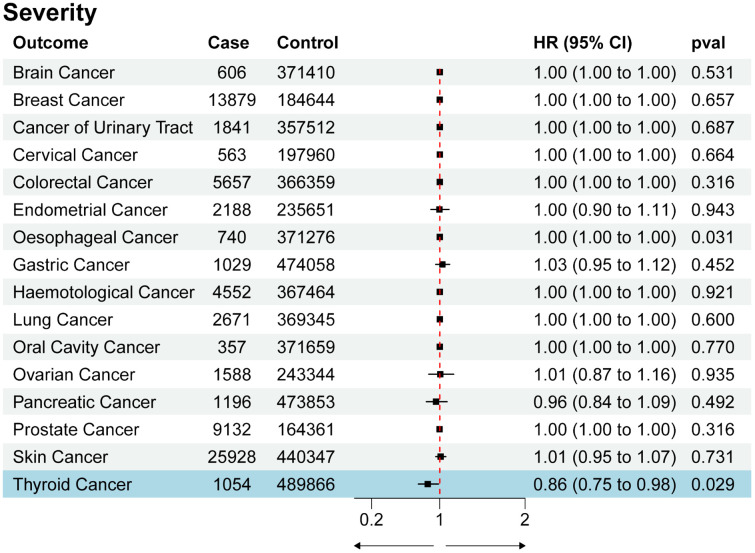
Forest plot of COVID-19 severity effects on multi-cancer risk.

**Figure 5:**
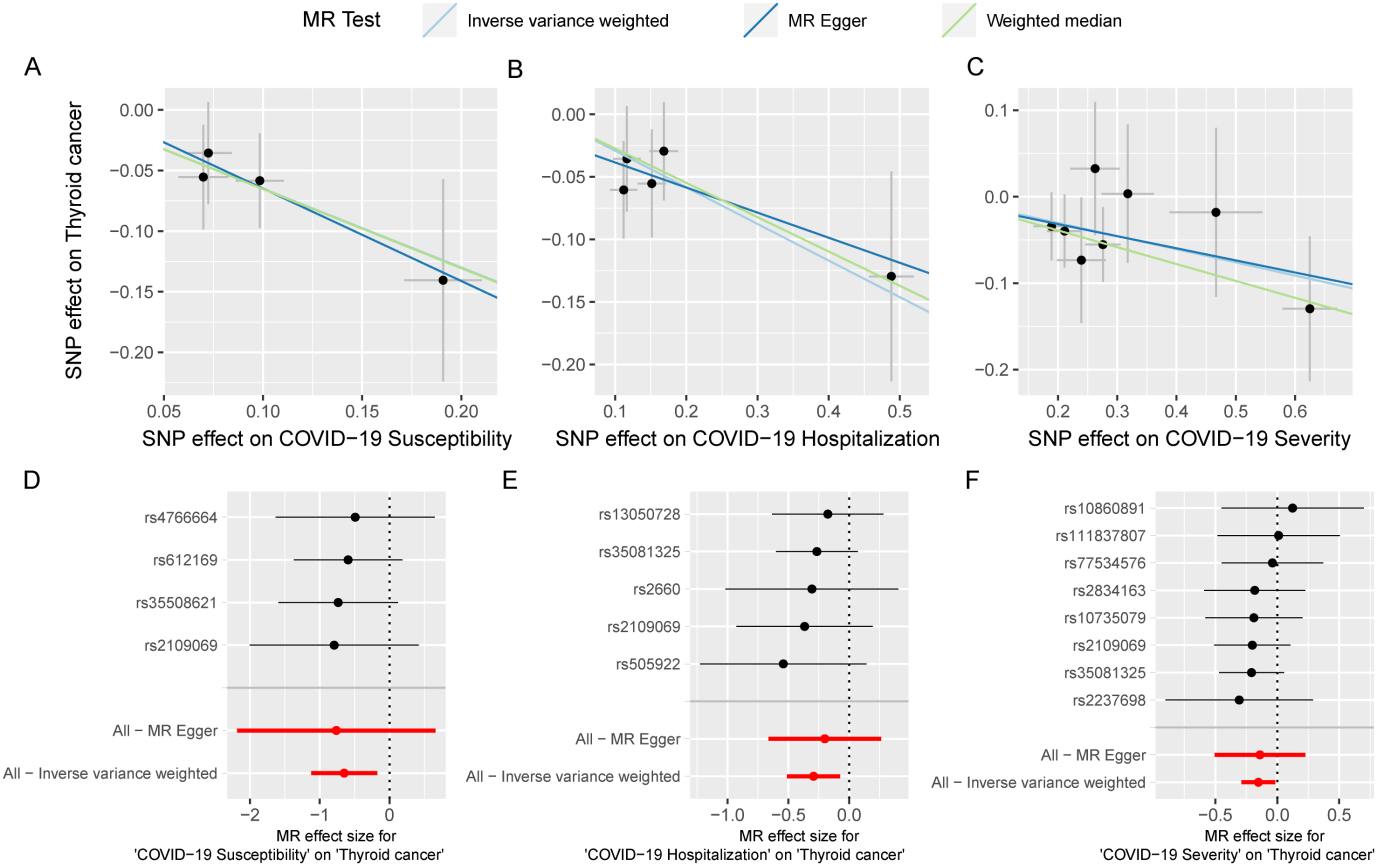
The MR analysis results between COVID-19 and multi-cancer risk are robust. scatter plot of casual estimate for MR test between COVID-19 susceptibility, hospitalization and severity on thyroid cancer **(A-C)**; forest plot of single SNP effect of COVID-19 susceptibility, hospitalization and severity on thyroid cancer **(D-F)**.

We utilized SNPs associated with COVID-19 susceptibility, hospitalization, and severity traits as exposure factors for mendelian randomization allocation. We assessed their impact on the risk of various cancers using the IVW, MR Egger, and Weighted Median methods (**Supplementary Table S4**). We found that COVID-19 susceptibility did not significantly influence the risk of various cancers, except for thyroid cancer (OR 0.52, 95% CI 0.33 to 0.84, p-value 0.007) (**Figure 2**). Additionally, intriguingly, the increase in the effects of COVID-19 hospitalization (OR 0.75, 95% CI 0.60 to 0.93, p-value 0.009) and severity (OR 0.86, 95% CI 0.75 to 0.98, p-value 0.029) was also associated with a reduced risk of thyroid cancer, while having no significant impact on other cancers (**Figures 3**, **4**). MR Egger and Weighted Median methods displayed results similar to IVW, although some of the results were not significant (**Figures 5A–C**). Estimating the effects of individual SNPs on thyroid cancer outcomes using the Wald ratio method, we found it was the collective effect of multiple SNPs, rather than individual SNPs, that influenced the outcome (**Figures 5D–F**). In summary, these findings suggest a clear genetic-level causal link between the increased risks of COVID-19 susceptibility, hospitalization, and severity and the reduced risk of thyroid cancer, as indicated by various methods. However, reverse MR analysis suggests that there is no reverse causal relationship between exposure and outcome (**Supplementary Table S5**).

### Sensitivity analysis

**Figure 6:**
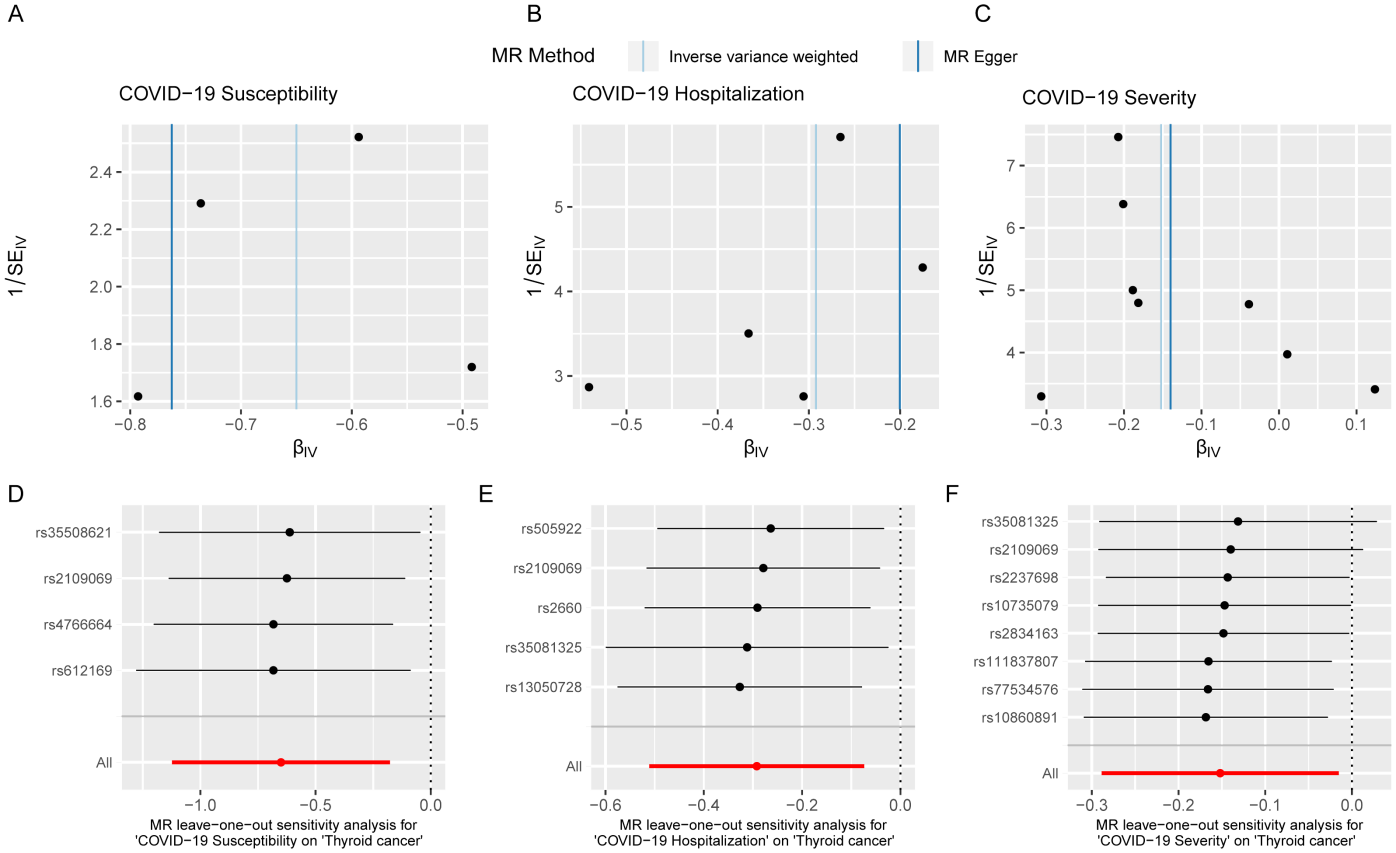
Sensitivity analysis indicates that the MR analysis was not affected by heterogeneity and pleiotropy. Funnel plot of single SNP effect **(A-C)**; forest plot of leave-one-out analysis **(D-F)**.

To validate the reliability of the analysis results, conducting sensitivity analysis is essential. We first used Cochran’s Q statistic to test the heterogeneity of the studies to avoid the impact of errors from non-experimental designs on the results. However, no heterogeneity was found to exist (**Supplementary Table S4**). Funnel plots showed symmetrical effect sizes around the point estimates of the exposure factors, suggesting no apparent pleiotropy (**Figures 6A–C**). Employing MR-PRESSO, we further scrutinized horizontal pleiotropy in our MR analysis. The non-significant p values (0.995, 0.998, 1) confirm the robustness of our findings in thyroid cancer research(**Supplementary Table S6**). Furthermore, the results of the leave-one-out analysis indicated that no individual SNP significantly influenced the outcomes, demonstrating the stability of the results (**Figures 6D–F**). In summary, the sensitivity analysis results indicate that the findings of this study are robust, free from interference caused by heterogeneity and pleiotropy.

## Discussion

Despite a wealth of evidence indicating that cancer patients face heightened risks of severe COVID-19 symptoms and increased mortality rates due to their compromised immune systems (26), limited research has been conducted on the potential cancer risk among individuals infected with COVID-19. The absence of standardized long-term care and health guidelines for COVID-19 patients, particularly concerning its impact on the occurrence of malignancies, contributes to our limited understanding in this area. We propose that this represents a matter of significant public health concern with universal implications.

To address this question, we employed two-sample Mendelian randomization (MR) to evaluate the association between COVID-19 susceptibility, hospitalization, and severity and the risk of human cancers. Our findings suggest that, among these three types of exposure to COVID-19, there is no significant effect on the incidence risk of most cancers. Despite SARS-CoV-2 being primarily a respiratory virus, exposure to it does not increase the odds ratio (OR) for lung cancer. However, an interesting exception is observed in the case of thyroid cancer in which both COVID-19 susceptibility, hospitalization, and severity are associated with a reduced risk (OR 0.52 for susceptibility, OR 0.75 for hospitalization, and OR 0.86 for severity). This suggests that genetic susceptibility to COVID-19 may have a potential protective effect on thyroid function and the risk of thyroid cancer, although the precise underlying mechanisms remain unclear.

The first question is why COVID-19 does not impact the incidence of most human cancers. However, our findings suggest that COVID-19 does not appear to raise the risk of cancer. One potential explanation is that the duration of SARS-CoV-2 infection and the symptoms of post-infection syndrome, although longer than typical acute viral infections, still constitute an acute process. This duration is still too brief when considering the extended period necessary for the accumulation of mutations required to initiate malignancy. Consequently, it is justifiable to conclude that COVID-19 may not have a significant influence on the risk of developing various cancers in the human body. Although studies have found that symptoms caused by SARS-CoV-2 infection can persist long-term, there is no substantial evidence reporting that the SARS-CoV-2 virus can persist in the human body long after infection, unlike carcinogenic viruses such as HPV that can persist in the body long-term. The transient nature of SARS-CoV-2 infection in the human body may physiologically reduce the risk of carcinogenesis.

Another key issue is why there is a negative causal relationship between the occurrence of thyroid cancer and exposure to COVID-19. A recent study has reported similar findings, although the results were not significant, possibly due to the significantly smaller sample size in the dataset used by the authors (174,995 vs. 490,920) (27). Experimental reports also support this conclusion; the SARS-CoV-2 virus does not seem to strongly attack the human thyroid (28, 29), despite the thyroid being one of the organs with abundant expression of ACE2 in the human body (30, 31). Moreover, pathological reports show that it is challenging to detect the presence of the SARS-CoV-2 virus in thyroid tissue after COVID-19 infection (32–34). This suggests that mechanistically, SARS-CoV-2 is unlikely to have an impact on thyroid carcinogenesis. One possible explanation for these phenomena is that they are related to genetic variations and immune surveillance mechanisms. Studies have indicated that COVID-19 infection can activate the immune system, including attacking immune cells, which may make the body more vigilant in monitoring and clearing potential cancer cells (35). We believe that individuals susceptible to COVID-19 have more pronounced symptoms, indicating an easily activated immune system. In this scenario, the activated immune system increases the detection of cancer cells, which can reduce the occurrence of thyroid cancer.

An interesting result is that as the severity of COVID-19 increases, the risk of various cancers decreases (the highest OR for COVID susceptibility and cancer risk is 1.48, while for COVID hospitalized it is 1.14, and for COVID severity it drops to 1.03), despite the results not being significant. And not only the overall trend of cancer decreases with increasing COVID-19 degree, but also the OR values for thyroid cancer follow this pattern. Another study also found similar patterns, which suggests that as the severity of COVID-19 increases, the relative risk of cancer in patients decreases (27). Based on this, we believe that this is unlikely to be a coincidence in the data, but rather a general trend. This is consistent with our speculation that carriers of genetic variations associated with more severe COVID-19 symptoms may have higher levels of immune surveillance, thereby possessing stronger abilities to eliminate malignant cells, leading to a lower likelihood of thyroid cancer occurrence. The severity of COVID-19 symptoms and the immune levels of patients after infection are related, which is also one of the reasons why younger patients typically have more severe symptoms. Research conclusions support this viewpoint; levels of cytokines and neutrophils increase in the bodies of severe COVID-19 patients (36). Therefore, we speculate that individuals susceptible to COVID-19 may have higher levels of innate immune activity, and the increased threshold of innate immunity not only leads to more pronounced symptoms during COVID-19 infection but also increases the probability of clearing cancerous cells in the body.

Despite providing evidence and revealing the connection between COVID-19 infection and various cancers, our study has some limitations. Firstly, the samples studied were predominantly from individuals of European descent, limiting the generalizability of the conclusions as the incidence rates of thyroid cancer vary across different countries (37). Secondly, The study included 16 types of cancer datasets available in public databases. However, these datasets do not encompass all known human cancers, and reliance on a single database source may introduce selection bias. Additionally, MR studies explain the impact of genetic variations on outcomes. However, in the real world, interference from environmental factors, lifestyle habits, and other acquired factors might not yield the same results.

## Conclusion

In conclusion, this study utilizes the Mendelian randomization (MR) method and incorporates comprehensive genome-wide association studies (GWAS) data to clarify that the susceptibility to COVID-19 does not have a significant impact on the risk of most cancers. However, our research emphasized a specific protective effect of COVID-19 against thyroid cancer. These findings are of great importance as they provide essential evidence for the development of effective screening strategies for patients with various types of cancer and enhance our comprehension of the association between COVID-19 and the risk of cancer in humans.

## Data Availability

The original contributions presented in the study are included in the article/**Supplementary material**. Further inquiries can be directed to the corresponding author.
